# Loss of conductance between mesophyll symplasm and intercellular air spaces explains nonstomatal control of transpiration

**DOI:** 10.1073/pnas.2504862122

**Published:** 2025-11-19

**Authors:** Piyush Jain, Sabyasachi Sen, Fulton E. Rockwell, Robert J. Twohey, Annika E. Huber, Sahil A. Desai, I-Feng Wu, Tom De Swaef, Mehmet M. Ilman, Anthony J. Studer, N. Michele Holbrook, Abraham D. Stroock

**Affiliations:** ^a^Sibley School of Mechanical and Aerospace Engineering, Cornell University, Ithaca, NY 14853; ^b^Kavli Institute at Cornell for Nanoscale Science, Cornell University, Ithaca, NY 14853; ^c^Department of Organismic and Evolutionary Biology, Harvard University, Cambridge, MA 02138; ^d^Department of Crop Sciences, University of Illinois Urbana-Champaign, Urbana, IL 61801; ^e^Smith School of Chemical and Biomolecular Engineering, Cornell University, Ithaca, NY 14853; ^f^Plant Sciences Unit, Flanders Research Institute for Agriculture, Fisheries and Food, 9820 Merelbeke, Belgium; ^g^School of Integrative Plant Science, Cornell University, Ithaca, NY 14853

**Keywords:** undersaturation, mesophyll, stomata, plasma membrane

## Abstract

The conventional assumption is that stomatal conductance ($g_{s}$) dominates the regulation of water and carbon dioxide fluxes between leaves and the atmosphere. Here, a nanoreporter of water status at the mesophyll cell surface and local xylem within intact maize leaves documents significant undersaturation of water vapor in the outside-xylem zone (OXZ) and a large loss of conductance of this zone ($g_{\mathrm{oxz}}$) at moderate xylem water stress, without stomatal closure or turgor loss. The ratio of the resistances $\left(1/g_{\mathrm{oxz}}\right)/\left(1/g_{s}\right)$ serves as a predictive phenotype of undersaturation, nonstomatal regulation of transpiration, errors in standard gas exchange analysis, and an increase of intrinsic water use efficiency (iWUE). Cell-scale access to water status reveals symplasmic-apoplasmic disequilibrium and informs a biophysical model that can explain experimental observations quantitatively based on localization of variable conductance to the plasma membrane. This work opens paths of inquiry into the molecular basis and functional consequences of nonstomatal regulation of transpiration.

## Significance

This research provides experimental and theoretical evidence that stomates are not the sole regulators of transpiration in plants. We use a nanoreporter of water potential (AquaDust) to document significant nonstomatal control of transpiration, with significant gains in water-use efficiency and large local disequilibrium within leaf tissue under moderate drought stress. With the methods and biophysical model introduced here, we quantitatively explain this nonstomatal control of water loss based on loss of conductance of plasma membranes in the leaf. These developments open paths to investigate this phenomenon and pursue its implications for the design of crops with high water-use efficiency and for our understanding of water stress responses across both agricultural and natural ecological contexts.

**Fig. 1.**
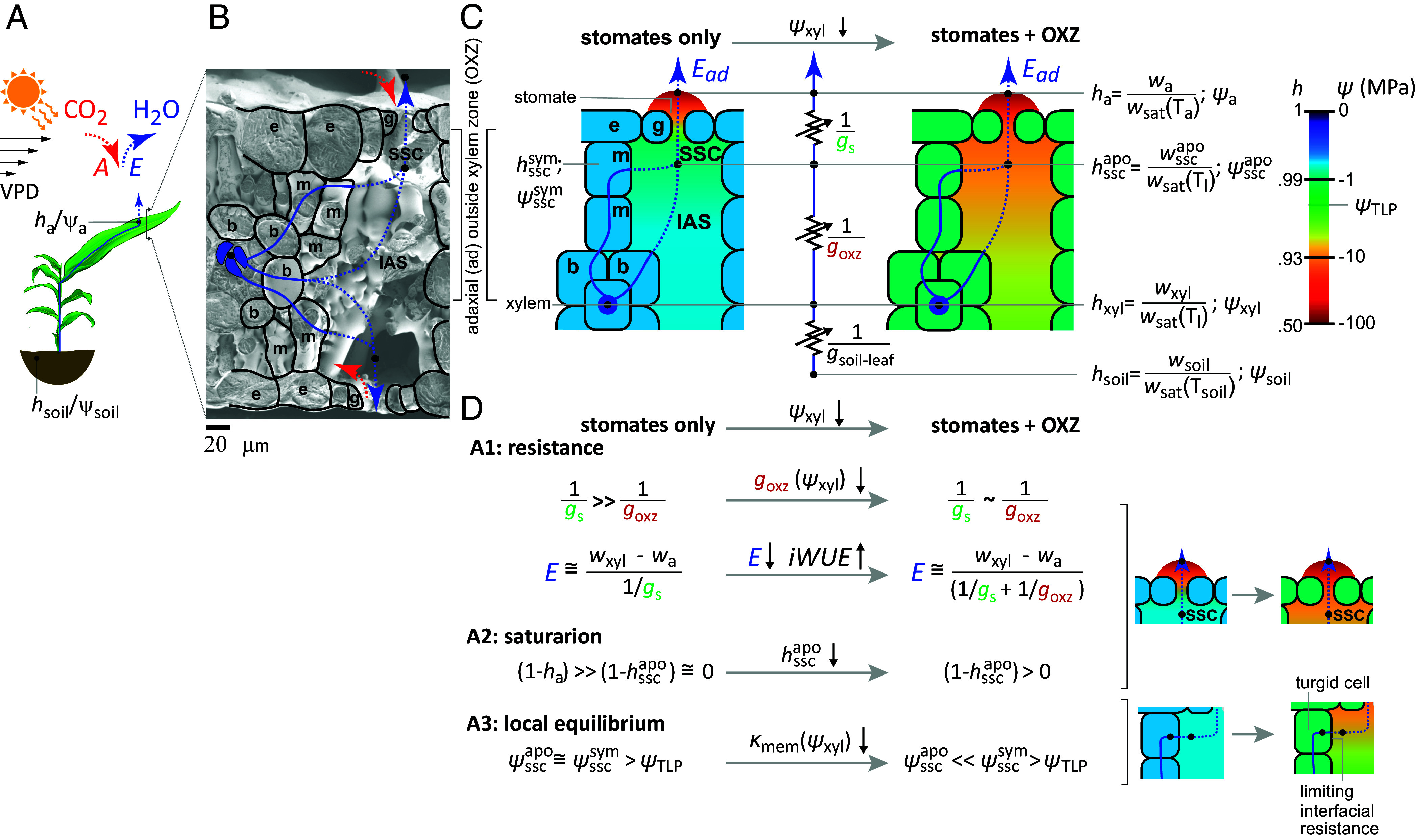
Stomatal and nonstomatal control of transpiration. (*A*) Schematic diagram of soil-plant-atmosphere continuum and gas exchange. The transpiration [E (mmol/m^2^/s)—blue curve] from high water status in the soil [relative humidity, $h_{\mathrm{soil}}≅1$ (–); water potential, $\psi_{\mathrm{soil}}≅0$ (MPa)] to the low water status in the atmosphere ($h_{a}<1$; $\psi_{a}\ll 0$) is actively regulated in response to environmental signals like soil water availability ($\psi_{\mathrm{soil}}$), thermal loading and light availability, and vapor pressure deficit between the leaf and atmosphere (VPD). This regulation also affects assimilation rates [A (mmol/m^2^/s)—red arrow]. (*B* and *C*) Outside xylem zone (OXZ) and stomates. Cryoelectron micrograph (*B*) and schematic diagrams (*C*) of cross-section of a maize leaf identifying xylem (blue shaded), bundle sheath (s), mesophyll (m), epidermal (e), and guard (g) cells, intercellular air spaces (IAS), substomatal cavity (SSC), and stomates. Water flows as a liquid (solid blue curves) through cell-to-cell paths and diffuses as a vapor (dashed blue curves) through the IAS, SSC, and stomates. On its path from the soil to the atmosphere we define three resistances [zigzag lines in (*C*)] in series: from soil to xylem in the leaf ($1/g_{soil-leaf}$), from the local xylem to the SSC apoplasm at water status $h_{\mathrm{ssc}}^{\mathrm{apo}}$ ($1/g_{\mathrm{oxz}}$), and from the SSC through stomates to the atmosphere ($1/g_{s}$), where $g_{soil-leaf}$, $g_{\mathrm{oxz}}$, $g_{s}$ are conductances per unit area of leaf (mmol/m^2^/s). Two scenarios are depicted in (*C*): the resistance of stomates alone control E (stomates only—*Left*) and the added resistances of the OXZ provides an additional, nonstomatal control of E (stomates + OXZ—*Right*). (*D*) Conventional assumptions about regulation of transpiration rate and their violation by large resistance in OXZ. A1: Stomates have been assumed to present the limiting resistance to transpiration ($1/g_{s}\gg 1/g_{\mathrm{oxz}}$); if the OXZ presents a resistance of the same order of magnitude ($1/g_{s}∼1/g_{\mathrm{oxz}}$) it will participate in limiting transpiration ($1/g_{\mathrm{oxz}}$ in denominator of expression for E on *Right*). A2: If stomates are limiting, the internal water status should remain near saturation ($1-h_{\mathrm{ssc}}^{\mathrm{apo}}≅0$—blue in diagram on *Right*); if the OXZ also limits flow, the inside of the leaf will become unsaturated ($1-h_{\mathrm{ssc}}^{\mathrm{apo}}>0$—orange in diagram on *Right*). A3: If the SSC remains saturated, then the local apoplasm and symplasm can remain near equilibrium without loss of cell turgor ($\psi_{\mathrm{ssc}}^{\mathrm{apo}}≅\psi_{\mathrm{ssc}}^{\mathrm{sym}}>\psi_{\mathrm{TLP}}$, where $\psi_{\mathrm{TLP}}$ is the water potential at the tugor loss point); if the SSC become significantly undersaturated, then large disequilibrium must exist between the apoplasm and symplasm ($\psi_{\mathrm{ssc}}^{\mathrm{apo}}\ll \psi_{\mathrm{ssc}}^{\mathrm{sym}}>\psi_{\mathrm{TLP}}$) and the interface between these domain must present a large resistance to water flow. The values of water status in the diagrams in *C* and *D* are color coded based on the color map in (*C*).

Leaves play a dominant role in the exchange of water (transpiration, *E*), carbon dioxide (net assimilation, *A*), and energy with the atmosphere (Fig. 1*A*) (1). Their ability to regulate this exchange in response to changing soil water availability, vapor pressure deficit (VPD), and other environmental parameters defines plant productivity, efficiency, and resilience (2). Incomplete understanding of these modes of regulation has hindered our ability to predict, manage, and modulate plant function in both ecological (3) and agricultural contexts (4).

In transpiration, water moves from the soil to the atmosphere through the plant along a gradient of water potential [ψ (MPa)] or equivalent relative humidity ($h=w/w_{\mathrm{sat}}$ where w and $w_{\mathrm{sat}}$ are the mole fraction of water vapor and its value at saturation) from the nearly saturated soil ($\psi_{\mathrm{soil}}≅0;\;h_{\mathrm{soil}}≅1$) to the undersaturated air in the atmosphere ($\psi_{a}\ll 0;\;h_{a}<1)$ (Fig. 1*A*). Within a leaf (Fig. 1 *B* and *C*), water passes from the xylem through the outside xylem zone (OXZ) formed of bundle sheath cells (b), mesophyll cells (m), and intercellular air spaces (IAS), to the substomatal cavities (SSC) upstream of the stomatal pores defined by guard cells (g) in the epidermis (e).

Three coupled assumptions define conventional understanding of the physiology that regulates gas exchange (Fig. 1*D*): A1—stomata present the limiting resistance [$1/g_{s}$ (1/(mmol/m^2^/s))] to, and so dominant point of control for, transpiration [E (mmol/m^2^/s)] and net assimilation [A (µmol/m^2^/s)] (5). This assumption implies that the hydraulic resistances associated with the entire upstream path from soil to the SSC are negligible relative to that of the stomates. With the inherent lack of selectivity of stomates to the outward diffusion of water vapor relative to the inward diffusion of carbon dioxide (CO_2_), this assumption also implies a significant constraint on the modulation of intrinsic water use efficiency (iWUE=A/E) via evolution (4), selection (6), or engineering (7) of stomatal properties. A2—If A1 holds, the inside of the leaf can remain close to saturation and one can assume that the deviation from saturation remains small relative to the deficit outside the stomates ($\left(1-h_{a}\right)\gg \left(1-h_{\mathrm{ssc}}^{\mathrm{apo}}\right)≅0$; $h_{\mathrm{ssc}}^{\mathrm{apo}}$ is the equivalent relative humidity in the apoplasm at the substomatal cavities). Importantly, in conventional analyses of gas exchange, this assumption is used to close the equations for water and CO_2_ fluxes to estimate stomatal conductance as, $g_{s}^{\mathrm{sat}}=E/\left(w_{\mathrm{sat}}\left(T_{l}\right)-w_{a}\right)$, where $w_{\mathrm{sat}}$ is the mole fraction of water vapor at saturation at the temperature of the leaf, $T_{l}$ [*SI Appendix*, section S3; LI-COR; (5)]. A3—If A2 holds, local equilibrium of water status can be maintained between the symplasm and apoplasm ($\psi_{\mathrm{ssc}}^{\mathrm{sym}}≅\psi_{\mathrm{ssc}}^{\mathrm{apo}}>\psi_{\mathrm{TLP}}$; $\psi_{\mathrm{ssc}}^{\mathrm{sym}}$ is the water potential in the symplasm of cells lining the substomatal cavities; $\psi_{\mathrm{TLP}}$ is the turgor loss point) without loss of turgor during active transpiration (i.e., when stomata are open). This assumption of local equilibrium underpins current models of water transport through the OXZ (8, 9) and stomatal regulation (10, 11).

Two early studies with gas exchange using inert gases (12) and leaves stripped of their epidermis (13) suggested the emergence of a non-negligible resistance within the OXZ, in violation of assumption A1. Other interrogations of the water relations of leaves using a combination of gas exchange measurements, cell probes, and leaf psychrometers (14, 15) or of the evaporative method and the pressure chamber (16–18) have been compatible with all three assumptions, without excluding possible violations of one or more of them (*SI Appendix*, sections S1 and S4). These studies with the pressure chamber (16–18), and studies by our team with the nanoreporter AquaDust (19, 20), reported loss of conductance of the OXZ with increasing leaf or xylem water stress without violation of A1. We performed our previous work in maize at low values of vapor pressure deficit relative to the leaf, VPD≅0.7 to 2 kPa (19). These studies also concluded that the resistance upstream of the leaves ($1/g_{soil-leaf}$) remains negligible, relative to stomata, for moderate drought. Recent measurements employing inline O^18^ discrimination with gas exchange (21–23), dual-sided gas exchange (24, 25), or both (26, 27) have again challenged these assumptions with inferences of significant undersaturation within transpiring leaves of a variety of species [(1 − *h_ssc_^apo^*)—violating A2], and the suggestion of disequilibrium between symplasm and apoplasm in the OXZ with the emergence of a limiting hydraulic resistance at the symplasm–apoplasm interface (violating A3). These studies report decreasing $h_{\mathrm{ssc}}^{\mathrm{apo}}$ with increasing VPD and suggested a mechanistic connection to transport through the plasma membrane (24, 25, 27). The macroscopic nature of these recent studies leave open alternative scenarios that could explain the directly measured processes without involving significant loss of tissue conductance (A1), undersaturation (A2), or symplasmic-apoplasmic disequilibrium (A3) (28, 29).

To test the validity of these assumptions and the consequences of their violation we use a nanoreporter, AquaDust, to access localized water status within the OXZ (A2), thereby assessing the conductance of the OXZ relative to that of the stomates (A1), and interrogating the state of symplasmic and apoplasmic equilibrium (A3) in intact, transpiring leaves (28–31). We then address the dependence of any observed nonstomatal modulation of gas exchange on water availability ($\psi_{\mathrm{xyl}}$) and demand (VPD) and derive a mechanistic, predictive framework based on physiological and physical processes in the OXZ.

## Results

**Fig. 2.**
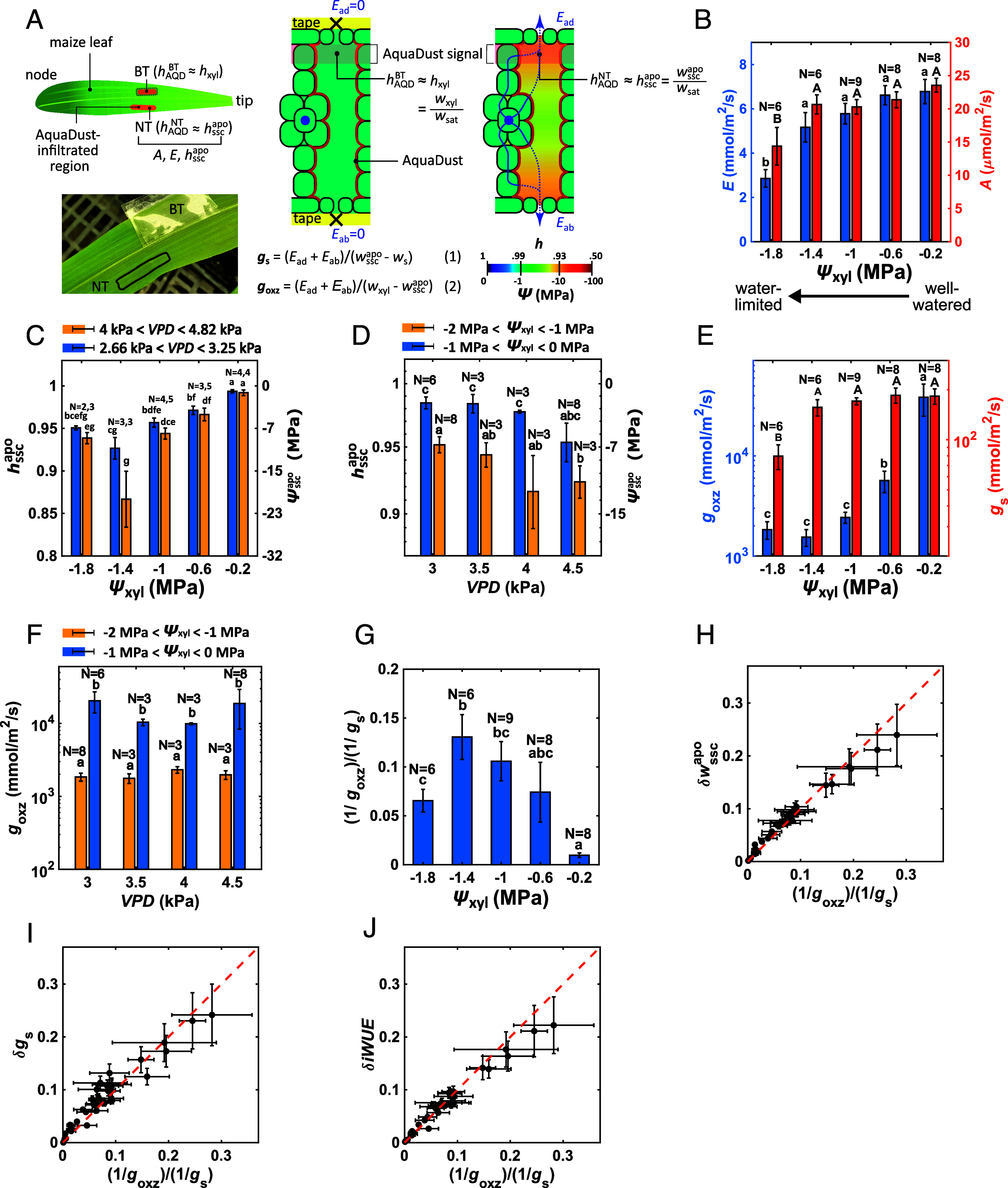
Direct measurement of undersaturation and nonstomatal control of transpiration by the OXZ. (*A*) Use of AquaDust to measure the water status upstream ($h_{\mathrm{xyl}}$ and $\psi_{\mathrm{xyl}}$) and downstream ($h_{\mathrm{ssc}}^{\mathrm{apo}}$ and $\psi_{\mathrm{ssc}}^{\mathrm{apo}}$) of the OXZ on intact leaves with gas exchange analysis. For a transpiring location on the leaf (labeled “NT” for “No-Tape”), the AquaDust signal reports $h_{\mathrm{ssc}}^{\mathrm{apo}}$; for a nontranspiring location on the leaf (labeled “BT” for “Both-sides Taped”), the SSC is in near equilibrium with the local leaf xylem such that the AquaDust signal reports $h_{\mathrm{xyl}}$. Gas exchange measurements on the NT region provide rates of transpiration [E = $E_{\mathrm{ad}}+E_{\mathrm{ab}}$ (mmol/m^2^/s)] and CO_2_ assimilation [A (μmol/m^2^/s)]. The conductances of the stomates (Eq. **1**) and OXZ (Eq. **2**) can be defined with these potentials without an assumption of internal saturation. See previous publications for detailed methods (19, 20). (*B*) Transpiration rates (E—blue bars, *Left* axis) and assimilation rates (A—red bars, *Right* axis) as a function of $\psi_{\mathrm{xyl}}$ from well-watered ($\psi_{\mathrm{xyl}}=-0.2\pm 0.2$ MPa) to near the TLP ($\psi_{\mathrm{xyl}}=-1.8\pm 0.2$ MPa). (*C* and *D*) Saturation of SSC with water availability ($\psi_{\mathrm{xyl}}$—*C*) and demand (VPD—*D*). AquaDust measurements of $h_{\mathrm{ssc}}^{\mathrm{apo}}$ (*Left* axis) and $\psi_{\mathrm{ssc}}^{\mathrm{apo}}$ (*Right* axis) as a function of $\psi_{\mathrm{xyl}}$ for low VPD (blue bars) and high VPD (yellow bars) (*C*) and as a function of VPD for high $\psi_{\mathrm{xyl}}$ (low stress—blue bars) and low $\psi_{\mathrm{xyl}}$ (high stress—yellow bars) (*D*). (*E*) Variations in $g_{\mathrm{oxz}}$ and $g_{s}$ as a function of $\psi_{\mathrm{xyl}}$. (*F*) Variations in $g_{\mathrm{oxz}}$ as a function of VPD for well-watered (blue bars) and water-limited (yellow bars) cases. (*G*) Ratio of OXZ hydraulic resistance (${1/g}_{\mathrm{oxz}}$) to stomatal resistance (${1/g}_{s}$) as a function of $\psi_{\mathrm{xyl}}$. (*H*–*J*) Ratio of resistances, [$\left({1/g}_{\mathrm{oxz}}\right)/\left(1/g\_s\right)$] as a predictive phenotype of impact of nonstomatal regulation of transpiration. Relative degree of undersaturation of SSC ($\delta w_{\mathrm{ssc}}^{\mathrm{apo}}=$ $\frac{w_{\mathrm{sat}}\left(T_{\mathrm{leaf}}\right)-w_{\mathrm{ssc}}^{\mathrm{apo}}}{w_{\mathrm{sat}}\left(T_{\mathrm{leaf}}\right)-w_{a}}$—*H*; r^2^ = 0.98), relative error in the conventional assessment of $g_{s}$ based on assumption of saturation in IAS ($\delta g_{s}=\frac{g_{s}-g_{s}^{\mathrm{sat}}}{g_{s}}$, where $g_{s}^{\mathrm{sat}}$ is the standard value of stomatal conductance assuming saturation of SSC—*I*; r^2^ = 0.93), and relative gain in intrinsic water-use efficiency [$\delta \left(iWUE\right)=\frac{iWUE-iWUE_{\mathrm{sat}}}{\mathrm{iWUE}}$, where $iWUE_{\mathrm{sat}}$ is the value assuming saturation of SSC—*J*; r^2^ = 0.97]. See *Results* and *SI Appendix*, section S3 for explanation and calculations. In frames b, c, e, and g, the measurements are binned in ranges of $\psi_{\mathrm{xyl}}$: 0 to -0.4, -0.4 to -0.8, -0.8 to -1.2, -1.2 to -1.6, and -1.6 to -2 MPa. In frames *D* and *F*, measurements are binned in ranges of VPD: 2.66 to 3.25, 3.25 to 3.75, 3.75 to 4.25, and 4.25 to 4.82 kPa. In frames *B*–*G*, N represents the number of biological replicates and statistically significant differences at the *P* < 0.05 level are labeled with different letters.

Here we focus on maize (*Zea mays L.* W22 inbred line) infiltrated with AquaDust (Fig. 2*A*). AquaDust exploits Förster Resonance Energy Transfer (FRET) between donor and acceptor dyes bound in a hydrogel matrix (19, 20, 32). Once infiltrated into a leaf, these nanoreporters coat the cell walls surrounding the IAS of the OXZ (red outline in Fig. 2*A*). AquaDust signal from leaf regions with obstructed transpiration (both sides taped—BT) provides an estimate of xylem water status upstream of the OXZ ($h_{\mathrm{xyl}}$ or $\psi_{\mathrm{xyl}}$), and signal from unobstructed transpiring leaf regions (no tape—NT) provides a direct measurement of the water status downstream of the OXZ at the SSC ($h_{\mathrm{ssc}}^{\mathrm{apo}}$ or $\psi_{\mathrm{ssc}}^{\mathrm{apo}}$) (19, 20). Importantly, the latter quantity provides a direct measure of the water vapor partial pressure in the leaf airspace interior to the stomata, allowing us to evaluate $g_{s}$ without assumption A2 of near saturation within the leaf (Eq. **1** in Fig. 2*A*) (5) and $g_{\mathrm{oxz}}$ (Eq. **2** in Fig. 2*A*) (19, 20). See *SI Appendix*, Sections S1–S3 and previous publications for details (19, 20). We note that measurements of water potentials with AquaDust below the turgor loss point (~–2 MPa) involve using our calibration curve beyond the range over which we can verify it in planta with the Schölander Pressure Chamber (32); the accuracy of AquaDust in this extended range is supported by ex situ calibration (*SI Appendix*, section S1 and Fig. S1).

### Undersaturation of Leaf Airspaces Evolves in Response to Both Xylem Water Status and VPD.

For 37 plants and 87 measurements, we varied xylem water status ($\psi_{\mathrm{xyl}}$) and evaporative demand relative to leaf (VPD) independently (*SI Appendix*, Fig. S2). With decreasing $\psi_{\mathrm{xyl}}$ for all values of VPD, the transpiration rate, E (mmol/m^2^/s) and net CO_2_-assimilation rate, A [μmol/m^2^/s] decreased but only fell significantly as $\psi_{\mathrm{xyl}}$ approached the turgor loss point ($\psi_{\mathrm{TLP}}$ ≅-2 MPa) (Fig. 2*B*). For intermediate values of $\psi_{\mathrm{xyl}}$ (−1.4 ± 0.2 MPa) we observe the emergence of significant internal undersaturation ($h_{\mathrm{ssc}}^{\mathrm{apo}}≅0.93$; $\psi_{\mathrm{ssc}}^{\mathrm{apo}}≅-10$ MPa) at both high and low VPD (Fig. 2*C*; blue bars vs. yellow bars). At the lowest value of $\psi_{\mathrm{xyl}}$ ($-1.8\pm 0.2MPa≅\psi_{\mathrm{TLP}}$), water status in the SSC recovered as the reduction of transpiration due to stomatal closure reduced the dynamic pressure drop across the OXZ. Undersaturation tended to increase modestly (lower apoplastic humidity) with increasing VPD across both ranges of xylem stress ($\psi_{\mathrm{xyl}}$, Fig. 2*D*; blue bars vs. yellow bars). Importantly, these direct measurements of undersaturation in the IAS using AquaDust (violation of assumption A2) confirm, qualitatively, previous assessments of this phenomenon in maize and other species using gas exchange methods (24, 25). Quantitatively, we have not observed values of $h_{\mathrm{ssc}}^{\mathrm{apo}}$ as low as those reported previously ($h_{\mathrm{ssc}}^{\mathrm{apo}}≅0.8$ in maize) (25). Exploration of this quantitative difference is a priority (30) and should yield interesting insights, for example, on the distribution of sites of evaporation and uptake of carbon dioxide within the OXZ (33). See *SI Appendix*, section S1 for further discussion on the difference between our method using AquaDust and those involving 2-sided gas-exchange and isotope discrimination.

### Decline in OXZ Conductance Evolves in Response to Xylem Water Status, not VPD.

Evaluating the outside-xylem zone conductance, $g_{\mathrm{oxz}}$ (Fig. 2*E*, blue bars) based on AquaDust (Fig. 2 *C* and *D*) and gas exchange (Fig. 2*B*) data, we observed a dramatic drop (25-fold) that starts well above $\psi_{\mathrm{TLP}}$. In contrast, $g_{s}$ (Fig. 2*E*, red bars) only decreased significantly (threefold) in the most stressed plants ($\psi_{\mathrm{xyl}}=-1.8\pm 0.2$ MPa). While $g_{\mathrm{oxz}}$ responded strongly to $\psi_{\mathrm{xyl}}$ (Fig. 2*E*), it showed no significant dependence on VPD (Fig. 2*F*), revealing that the effects of VPD on $h_{\mathrm{ssc}}^{\mathrm{apo}}$ (Fig. 2*D*) are dynamic, resulting from an increase in the flux with increasing VPD rather than a change in OXZ conductance. We observed these same trends in $h_{\mathrm{ssc}}^{\mathrm{apo}}$ and $g_{\mathrm{oxz}}$ as a function of $\psi_{\mathrm{xyl}}$ in four other inbred genotypes in maize (*SI Appendix*, section S5 and Figs. S4–S6) and in a C3 species (Vicia faba L.; *SI Appendix*, section S6 and Fig. S7).

### The Ratio $g_{s}/g_{oxz}$ Captures the Degree of Undersaturation and Its Effects On Gas Exchange.

To clarify the importance of declines in $g_{\mathrm{oxz}}$ for regulation of gas exchange, in Fig. 2*G*, we plot the ratio of the resistances of the OXZ and the stomates: $\left(1/g_{\mathrm{oxz}}\right)/\left(1/g_{s}\right)=g_{s}/g_{\mathrm{oxz}}$. In well-watered maize ($\psi_{\mathrm{xyl}}=-0.2\pm 0.2$ MPa), the stomatal resistance was completely dominant ($g_{s}/g_{\mathrm{oxz}}≅0.01$) as conventionally assumed (A1—Fig. 1*D*). At intermediate stresses ($\psi_{\mathrm{xyl}}=-0.6$ to -1.4 MPa) for which $g_{s}$ remained high as $g_{\mathrm{oxz}}$ dropped (Fig. 2*E*), the ratio grew, such that the resistance of the OXZ made a non-negligible contribution to the total resistance to transpiration ($g_{s}/g_{\mathrm{oxz}}≅0.15)$. With the closure of the stomates at the most stressed conditions ($\psi_{\mathrm{xyl}}=-1.8\pm 0.2$ MPa), the importance of the OXZ resistance diminished ($g_{s}/g_{\mathrm{oxz}}≅0.06$).

To test the value of this ratio as a predictive phenotype of nonstomatal regulation of transpiration, we plot relative degree of undersaturation in internal water status (${\delta w}_{\mathrm{ssc}}^{\mathrm{apo}}=\frac{\left(w_{\mathrm{sat}}\left(T_{\mathrm{leaf}}\right)-w_{\mathrm{ssc}}^{\mathrm{apo}}\right)}{\left(w_{\mathrm{sat}}\left(T_{\mathrm{leaf}}\right)-w_{a}\right)}$—Fig. 2*H*), relative error in conventional gas exchange analysis based on assumption A2 ($\delta g_{s}=\frac{g_{s}-g_{s}^{\mathrm{Gaastra}}}{g_{s}}$ —Fig. 2*I*), and relative gain in iWUE ($\delta \left(iWUE\right)=\frac{iWUE-iWUE^{\mathrm{sat}}}{\mathrm{iWUE}}$ —Fig. 2*J*) as a function of $g_{s}/g_{\mathrm{oxz}}$ for all biological replicates in the maize inbred W22 (See *SI Appendix*, Note S3 for definitions). We note the strong, linear correlation of all of these signatures of nonstomatal regulation with this metric across all values of $\psi_{\mathrm{xyl}}$ and VPD tested. We find a further confirmation of the predictive value of $g_{s}/g_{\mathrm{oxz}}$ in our measurements in tomato (*SI Appendix*, Fig. S8) and cotton (*SI Appendix*, Fig. S9): $g_{s}/g_{\mathrm{oxz}}$ remained small (0.015 in tomato and cotton—*SI Appendix*, Figs. S8 *D* and S9 *D*), such that even as $g_{\mathrm{oxz}}$ dropped several fold as the plants passed from their well-watered states to near $\psi_{\mathrm{TLP}}$, no substantial undersaturation occurred (*SI Appendix*, Figs. S8*A* and S9*A*).

The consequences of this nonstomatal control are quantitatively important, as has been noted before (21–27). For the largest values of $g_{s}/g_{\mathrm{oxz}}$ the errors in the conventional estimate of $g_{s}$ can be as high as 24% (Fig. 2*I*). As a test of the appropriateness of this assessment of error, in *SI Appendix*, Fig. S10, we show that the values of $\delta g_{s}$ based on AquaDust (Fig. 2*I*) agree well with those based on a carbon balance using chlorophyll fluorescence (34). We also observe that the error in iWUE can grow to ~22% as the internal limitation on transpiration grows relative to that of the stomates (Fig. 2*J*). This observation suggests that the underlying processes that lead to a drop in $g_{\mathrm{oxz}}$ act in a selective manner on the flux of water relative to that of CO_2_, as indicated in previous reports (24, 25).

### Undersaturation Is Accompanied By Disequilibrium Between Symplasm and Apoplasm.

**Fig. 3.**
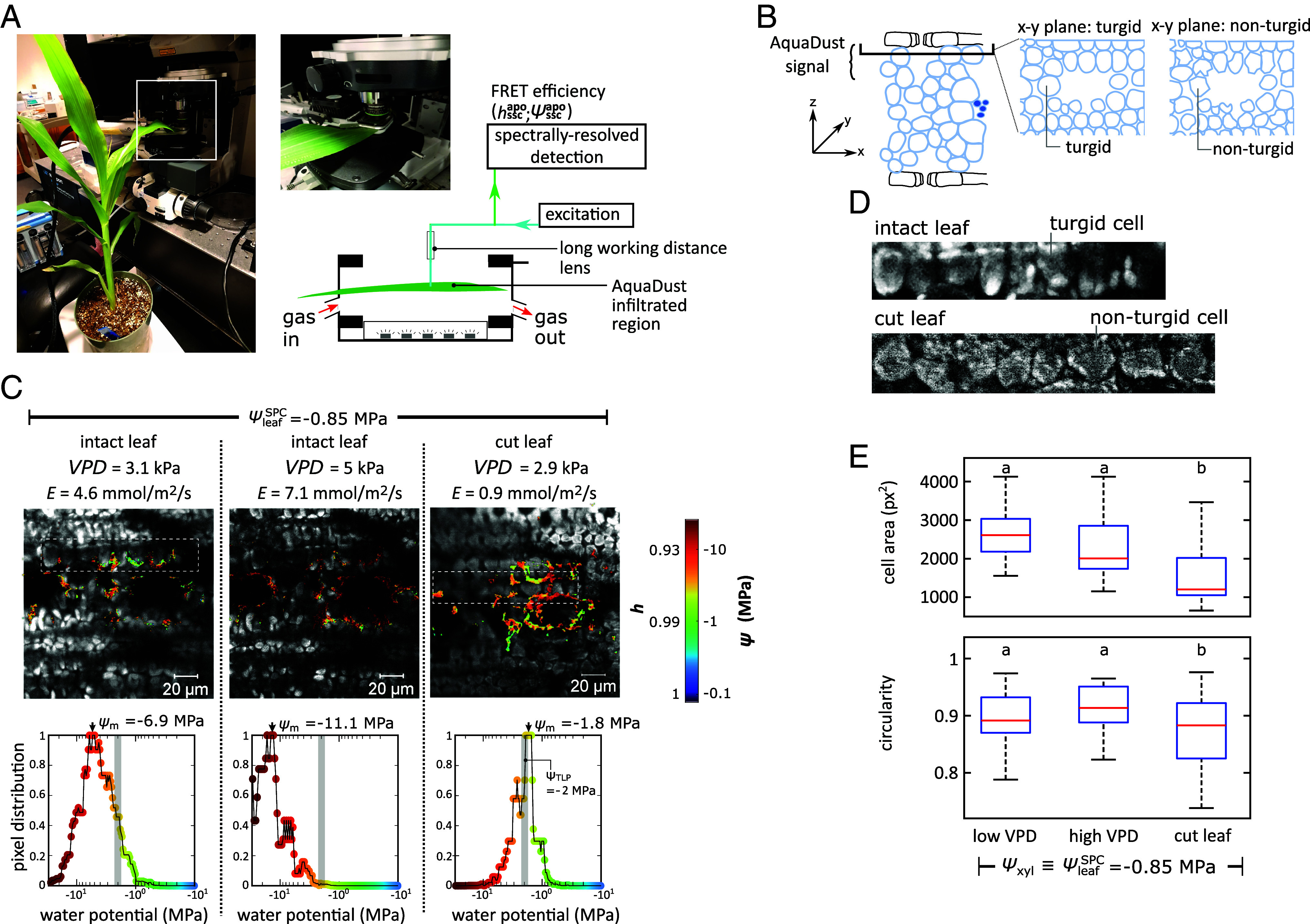
Extreme local gradients of water potential between mesophyll symplasm and SSC. (*A*) Spectrally resolved confocal imaging within a gas exchange cuvette in which a section of intact leaf infiltrated with AquaDust is clamped. The native fluorescence of the tissue and a pixel-wise assessment of FRET efficiency [ζ(ψ)] are captured at a depth of z≅24 μm. (*B*) Schematic diagrams indicating the location of the confocal section on a vertical cross-section (x−z—*Left*) and two examples of the cellular structure in the captured horizontal cross-sections (x−y—*Right*): turgid and turgor-loss states. (*C* and *D*) Horizontal confocal sections (x−y) of fluorescence and AquaDust FRET and pixel-level distribution of water potential (*C*) for three experimental conditions applied to a section of leaf of a maize *slac1-2* null mutant in the gas exchange cuvette: water limited ($\psi_{\mathrm{leaf}}^{\mathrm{SPC}}$ = −0.85 MPa) for low VPD (3.1 kPa), high VPD (5 kPa), and after excision of leaf (VPD = 2.9 kPa). Here, micrographs present AquaDust-reported water potential maps (color map on *Right*) overlaid on intensity of the 686 to 695 nm-channel in which chloroplasts are visible. Expanded views of a file of cells show turgid and turgor-loss states with intensity in the 686 to 695 nm-channel (*D*). In (*C*), histograms present of pixel-level values of water potential extracted from the images above, with the gray shaded bars representing the bulk tissue water potential measured using a Schölander Pressure Chamber ($\psi_{\mathrm{leaf}}^{\mathrm{SPC}}$). (*E*) Cell area and circularity for the three cases with the Tukey HSD test (n > 20 for each case and *P* < 0.05 for significant difference) using cell outlines. For intact leaf cases, mesophyll cells remain smooth and rounded [*Left* expanded view in (*D*)], indicating the maintenance of turgor even as AquaDust-reported water potentials in the mesophyll cell wall descended to a mode value, $\psi_{m}≅-11.1$ MPa, well below the overall water potential of the tissue, $\psi_{\mathrm{leaf}}^{\mathrm{SPC}}≅-0.85$ MPa. Upon excision from plant, the cells shrank and lost their smooth, rounded shape, indicating loss of turgor [*Right* expanded view in (*D*)] and AquaDust-reported water potentials recovered to a mode value, $\psi_{m}≅-1.8$ MPa, near the turgor-loss point of maize (TLP ≅ −2 MPa). See *SI Appendix*, section S8 for methods and additional confocal data.

Motivated by observation of large degrees of undersaturation in apparently turgid leaves, we combined confocal microscopy (Zeiss LSM880; Olympus LMPlanFL N 20x/0.4 objective) with AquaDust to create maps of water status with cell-scale resolution within the mesophyll of intact leaves in the cuvette of a gas exchange system (CIRAS-3, PPSystems) (Fig. 3*A*). In order to maintain robust levels of transpiration under the low light levels required for imaging, measurements were performed on a maize mutant (*slac1-2* null in W22 inbred background) in which stomates remain open except at turgor loss (35). Confocal sections (*x*-*y* planes—Fig. 3*B*) of tissue autofluorescence and AquaDust emission were collected to assess pixel-level water status. See *SI Appendix*, section S8.

In a leaf with moderate xylem stress (transpiring leaf, $\psi_{\mathrm{leaf}}^{\mathrm{SPC}}$ = −0.85 MPa), AquaDust measurements of mesophyll cell wall water status from pixels surrounding the cells adjacent to the substomatal cavities (elliptically shaped dark zones) showed a shift toward lower pixel-level water potential (redder color; left-shifted histogram) as VPD and E increased (VPD = 3.1 to 5 kPa and *E* = 4.6 to 7.1 mmol/m^2^/s) (Fig. 3*C*). Notably, the mode values of the distributions of pixel-level water potentials for both values of VPD ($\psi_{m}$ = −6 and −11 MPa) indicate strong undersaturation, qualitatively consistent with our macroscopic measurements as presented in Fig. 2*C*. Upon cutting the leaf, the transpiration rate decreased with stomatal closure, and the pixel-level values of water potential shifted toward higher potentials, with $\psi_{m}$ approaching the range expected for the turgor loss potential, $\psi_{m}≅-1.8$ MPa. See *SI Appendix*, section S8 and Figs. S14–S18.

In the expanded views of the cells’ autofluorescence in Fig. 3*D*, we note a qualitative change in the shape of the cells between the intact (high VPD—left) and cut (right) states, with smooth, rounded edges for the intact case and more faceted surfaces in the cut case. Quantitatively, we found a significant drop in both size and circularity of cells between the intact leaf and cut leaf cases (Fig. 3*E* and See *SI Appendix*, section S8 for details) which we interpret as evidence of the presence and loss of turgor respectively. This interpretation is consistent with macroscopic observations of turgidity and the high stomatal conductance of the intact leaves, and the onset of leaf rolling and reduced transpiration in the cut case (*SI Appendix*, Fig. S17). Taken together, the cell-scale documentation of strong undersaturation directly surrounding turgid cells in intact leaves undergoing steady transpiration (Figs. 3 *C*–*E*) provides direct evidence for the violation of assumption A3 of local equilibrium: a large drop in water potential occurred between the turgid symplasm ($\psi_{\mathrm{sym}}>\psi_{\mathrm{TLP}}≅$ −2 MPa) and the exterior of the cell wall at which AquaDust reports $\psi_{\mathrm{ssc}}^{\mathrm{apo}}$ < −10 MPa (Fig. 3*C*). In other words, nearly the entire potential drop from xylem to the sites of evaporation occurred in the passage of water across plasma membrane and cell wall as inferred previously (24–26, 30); one or both of these components must present the dominant resistance to the transpiration stream in the OXZ. As discussed below and in *SI Appendix*, section S10, these observations also place constraints on the hydraulic properties of the symplastic path through the OXZ.

### Modeling Undersaturation Selects From Among Candidate Hydraulic Architectures.

**Fig. 4.**
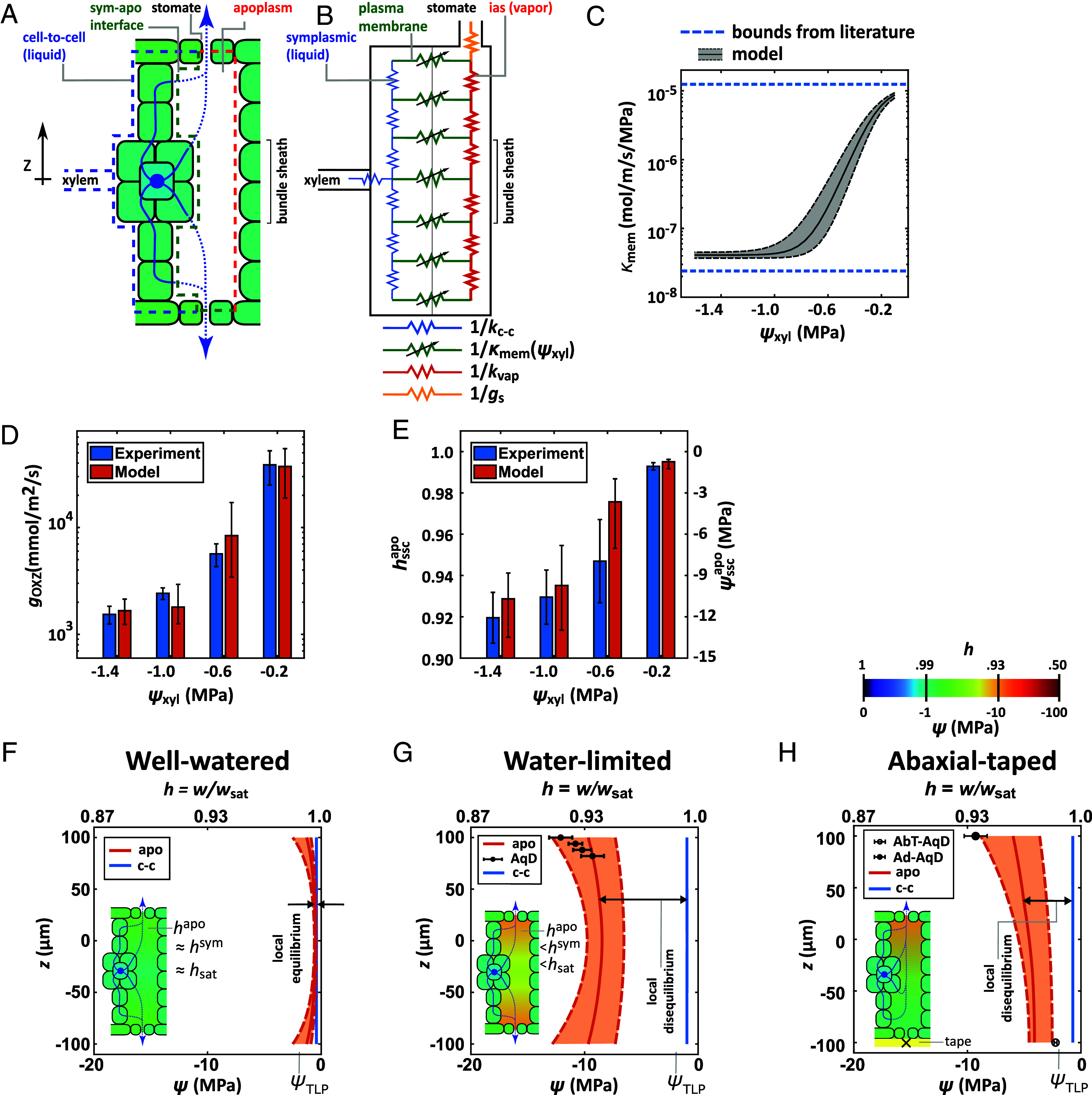
Model of leaf water transport in Outside-Xylem Zone with large loss of conductance in plasma membrane. (*A* and *B*) Two-compartment, pseudo-one-dimensional, continuum, steady-state model of water flow through OXZ introduced here for the hypothetical scenario in which the plasma membrane at the interface between the cell-to-cell and apoplasmic paths presents a limiting, variable conductance, $\kappa_{\mathrm{mem}}$. The cell-to-cell path (blue dashed outline; blue resistors in (*B*) defined by $1/k_{c-c}$) is coupled to the apoplasmic path [red-dashed outline in (*A*); red resistors in (*B*) defined by $1/k_{\mathrm{apo}}$] through the plasma membrane [green dashed line in (*A*); green resistors in (*B*) defined by $1/\kappa_{\mathrm{mem}}$]; the apoplasm feeds into a stomatal resistance [orange resistor in (*B*) defined by $1/g_{s}$]. Here, the hydraulic conductances, $k_{c-c}$ and $k_{\mathrm{apo}}$ (mol/m/s/MPa) and vapor conductance, $g_{s}$ (mmol/m^2^/s) are defined per unit area of leaf; the hydraulic conductance, $\kappa_{\mathrm{mem}}$ (mol/m/s/MPa) is defined per unit area of the symplasm–apoplasm interface. (*C*) Hypothetical functional dependence of conductance of plasma membrane, $\kappa_{\mathrm{mem}}$ on $\psi_{\mathrm{xyl}}$ used in the model presented in (*B*) for predictions in (*D*–*H*). The *Upper* and *Lower* bounds on $\kappa_{\mathrm{mem}}$ are from experiments on maize leaf protoplasts (29, 36). (*D* and *E*) Comparison of predictions from model based on variable conductance in (*C*) with experimentally observed $g_{\mathrm{oxz}}$ (*D*) and $h_{\mathrm{ssc}}^{\mathrm{apo}}$ (*E*) and as a function of $\psi_{\mathrm{xyl}}$ for VPD=3.8±0.2 kPa (mean VPD ± SE for data in Fig. 2). (*F*–*H*: Through-leaf profiles of water potential, ψ(z) (*Bottom* axes) and h(z) (*Top* axes—approximating linear dependence of h with psi) in cell-to-cell path ($\psi_{c-c}$—blue curve) and apoplasm ($\psi_{\mathrm{apo}}$—orange curve) predicted for well-watered ($\psi_{\mathrm{xyl}}$ = −0.4 ± 0.15 MPa—*F*), water-limited ($\psi_{\mathrm{xyl}}$ = −0.85 ± 0.15 MPa; VPD=4.2 kPa—*G*), and water-limited with obstructed transpiration from the adaxial surface ($\psi_{\mathrm{xyl}}$ = −1.2 MPa ± 0.15 MPa; VPD=4.2 kPa—*H*). Insets show schematic representations of profiles with colormap (*H*), with near equilibrium maintained between symplasm and apoplasm at low stress (both blue, near saturation—*F*) and disequilibrium between cell-to-cell path (blue) and apoplasm (green) at moderate stress (*G* and *H*). In both cases, $\psi_{c-c}$ remains above the turgor loss point (TLP ≅ −2 MPa), consistent with data presented in Fig. 3. In *G* and *H*, predicted profiles in apoplasm match measurements made with AquaDust in confocal sections (filled circles in adaxial SSC in *G* and *H*; open circle in abaxial SSC in *H*—see *SI Appendix*, section S11 for additional details).

To gain further insights into the physiological basis of these observations, we present a pseudo-one-dimensional model (Fig. 4*A*) of isothermal flow of water through the OXZ in which we can account for a cell-to-cell compartment (blue dashed line), an apoplasmic compartment (red dashed line), the coupling of these compartments through an interface defined by the plasma membrane and cell wall (green dashed line), and the downstream path through stomata. In *SI Appendix*, section S10, we derive this model and use it to explore four scenarios informed by hypotheses in the literature. We show that, when encoded in our modeling framework, the predictions of three of these hypotheses are incompatible with our observations. We present a compatible scenario that predicts the disequilibrium observed in Fig. 3 based on a hypothesis that the plasma membrane presents the limiting, variable resistance within the OXZ, as depicted in Fig. 4*B* (24, 25). See *SI Appendix*, Figs. S21–S23 and Table S6 for comparison of modeled scenarios.

The scenario that best fits the observed data includes the following (Fig. 4*B*): i) a variable interfacial resistance (green zigzag line with cross arrow indicating variability) dominated by the conductance of the plasma membrane with dependence on the upstream water potential [$\kappa_{\mathrm{mem}}(\psi_{\mathrm{xyl}})$ (mol/m/s/MPa) per interfacial area]; ii) a cell-to-cell resistance dominated by the conductivity of plasma membranes or plasmodesmata or both (*SI Appendix*, section S10) [$k_{c-c}$ (mol/m/s/MPa) per leaf area]; and iii) an axial apoplasmic resistance dominated by vapor conductivity [$k_{\mathrm{vap}}$ (mol/m/s/MPa) per leaf area] (*SI Appendix*, section S19). We model $\kappa_{\mathrm{mem}}\left(\psi_{\mathrm{xyl}}\right)$ as a sigmoid that respects the bounds on the hydraulic conductance of plasma membrane from experiments performed on maize leaf protoplasts (dashed blue lines; Fig. 4*C*) (29, 36). In Fig. 4*D*, we present predictions of this model for $g_{\mathrm{oxz}}$ with a range of values informed by the literature for fixed values $k_{c-c}$ and $k_{\mathrm{vap}}$ and $g_{s}$ [180 (mmol/m^2^/s)] and imposed VPD (3.8 ± 0.2 kPa—mean value of VPD in Fig. 2 data). We adjusted the parameters of $\kappa_{\mathrm{mem}}(\psi_{\mathrm{xyl}})$ to provide a best fit to measured $g_{\mathrm{oxz}}$ for these conditions (Fig. 4*D*; See *SI Appendix*, section S10 for details and *SI Appendix*, Tables S1–S5 for parameter values). The corresponding predictions of internal $h_{\mathrm{ssc}}^{\mathrm{apo}}$ are compatible with our measurements (Fig. 4*E*), providing a first quantitative check of the appropriateness of our model.

For well-watered conditions (Fig. 4*F*—$\psi_{\mathrm{xyl}}=-0.4\pm 0.15$ MPa) for which $\kappa_{\mathrm{mem}}$ is high, the symplasmic and vapor paths remain at or near equilibrium, with $\psi^{\mathrm{apo}}≅0$ ($h^{\mathrm{apo}}≅1$) throughout. For a water limited condition that corresponds to the experiment in Fig. 3*C* (Fig. 4*G*—$\psi_{\mathrm{xyl}}≅$ $\psi_{\mathrm{leaf}}^{\mathrm{SPC}}=-0.85\pm 0.15$ MPa), our preferred model predicts strong symplasmic-apoplasmic disequilibrium throughout the thickness and deep undersaturation near the stomates ($\psi_{\mathrm{ssc}}^{\mathrm{apo}}≅$ −12 MPa; $h_{\mathrm{ssc}}^{\mathrm{apo}}$ ≅ 0.92); this case approaches the limiting behavior suggested by Wong et al. (24) in which the dominant drop in potential occurs across the plasma membrane and through-leaf gradients in the IAS are minimal. For a case with obstructed transpiration from the abaxial surface (abaxial taped, AbT$-\psi_{\mathrm{xyl}}≅-1.2\pm 0.15$ MPa), the model predicts a monotonic gradient in the vapor from the nontranspiring abaxial surface ($E_{\mathrm{ab}}=0$) to the transpiring adaxial surface ($E_{\mathrm{ad}}>0$) accompanied by strong disequilibrium (Fig. 4*H*). Importantly, the predicted disequilibrium in both cases (Figs. 4 *G* and *H*) is compatible with the maintenance of high symplasmic water potential and the maintenance of turgid mesophyll cells adjacent to deeply undersaturated vapor. Furthermore, the predicted water potential profiles match confocal-based AquaDust measurements at multiple depths within the leaf (Fig. 4*G*) and macroscopic AquaDust measurements collected at the adaxial and abaxial surfaces (Fig. 4*H*), providing a second quantitative check of the appropriateness of the preferred model.

## Discussion

Our measurements and model elucidate the physiology underlying the emergence of nonstomatal control of transpiration based on loss of conductance in the OXZ. The direct measurement of undersaturation in Fig. 2 and of symplasm–apoplasm disequilibrium in Fig. 3 inform the localization of water potential gradients inside leaves, imposing important constraints on existing models of leaf hydraulics (8, 9) and stomatal responses (10, 11).

The identification of the ratio ($1/g_{\mathrm{oxz}}$)/($1/g_{s}$) as a predictive phenotype for undersaturation, errors in conventional calculations of $g_{s}$, and favorable variations in iWUE creates opportunities to pursue implications for ecophysiological function and the development of water-use efficient crops.

Based on access to water status at the cellular scale within leaves and a model of water movement through the OXZ, we conclude that our observations—symplasmic-to-IAS disequilibrium and variations in $g_{oxz}$—can be explained qualitatively and quantitatively with a hypothesis of variable conductance of the plasma membrane at the interfaces of both bundle sheath and mesophyll cells with the IAS and with parameter values measured independently (29, 36). Within this same modeling framework, our observations cannot be explained by variations in the conductance of the cell wall with reasonable parameter values (37). Notably, even full invasion of a desiccating cell wall with air cannot explain the significant disequilibrium shown in Fig. 3; the added radial resistance presented by an air-filled layer of ~200 nm thickness is negligible (see *SI Appendix*, Fig. S22). Moreover, were the presence of a material such as suberin in the cell wall responsible for the large disequilibrium observed in Fig. 3*C*, this disequilibrium would also be expected in well-watered conditions, which is not what we observe (see *SI Appendix*, Fig. S14). Further, our predictions based neither on variations in the conductance of the symplasm without loss of turgor (8) (*SI Appendix*, Fig. S21) nor on localized loss of conductance of the plasma membranes of just the bundle sheath (38) (*SI Appendix*, Fig. S23) are compatible with our observations (see *SI Appendix*, section S10 for additional discussion of scenarios). While our observations are compatible with predictions for the hydraulic architecture that involves loss of plasma membrane conductance in the mesophyll and bundle sheath, we encourage continued experimentation to challenge this hypothesis, as new data could support alternative conclusions.

This localization of the loss of conductance to the plasma membrane would resolve important questions about the physiological viability of internal undersaturation (30) and open a path to identify the molecular basis of this stress response, with aquaporins as an obvious starting point (*SI Appendix*, sections S12 and S13 and Figs. S25 and S26). The observation that loss of conductance in the OXZ occurs before the loss of stomatal conductance suggests a regulatory mechanism to allow for the control of water loss without immediately hindering CO_2_ assimilation, a phenomenon whose functional significance has been previously discussed (19, 20, 24–26, 30). Together, these methods, measurements, and models provide a foundation for the necessary reformulation of our existing understanding of leaf gas exchange (assumptions A1-A3) to account for active, nonstomatal management of water-use.

## Methods

### AquaDust Synthesis and Use.

AquaDust nanoparticles (70 to 100 nm diameter) were formed of hydrogel containing two matrix-bound fluorescent dyes enabling FRET-based sensing of water potential, as described previously (32). To measure local water potential in maize leaf mesophyll, 20 μL of AquaDust suspension was injected into leaves one day prior to measurement with a syringe pressed gently against the surface of the leaf. Successful injections were visually confirmed by a dark green discoloration spanning approximately 2 cm^2^ of the leaf tissue, a sign that the AquaDust suspension had permeated the leaf air spaces. The delay before measurement allowed the aqueous buffer to evaporate. To ensure the accuracy of water potential data and mitigate any potential impact of tissue damage from the injection, measurements were always performed at least 1 cm away from the injection site. Local water potentials were then determined by recording the fluorescence spectrum from the injection site. *SI Appendix*, section S1 presents details of the synthesis, characterization, injection protocol, calibration, and simultaneous measurement of water potential and gas exchange.

### Plant Material and Growing Conditions.

Maize (*Zea mays* L. W22 inbred line) plants were grown in a greenhouse under controlled conditions (28 ± 2 °C d/20 ± 2 °C night temperature, 40% RH day/80 to 90% RH night) with natural sunlight supplemented to maintain minimum PAR of 1,000 μmol m^−2^ s^−1^ during a 16 h photoperiod. Plants were grown in potting medium with slow-release fertilizer and measurements were conducted on uppermost newest, fully expanded, leaves at the V9-V10 vegetative growth stage. Additional genotypes and species used for comparative measurements were grown under identical conditions (see *SI Appendix*, section S2 for details).

### Measuring OXZ Conductance and Corrected Stomatal Conductance With AquaDust in a Transpiring Maize Leaf.

In order to calculate the hydraulic conductance of the outside-xylem zone ($g_{\mathrm{oxz}}$) and the stomatal conductance to water ($g_{s}$), we measured gas exchange variables [CO_2_ assimilation rates (A), transpiration rates (E), etc.] followed by measurements of local leaf water potential (ψ) with AquaDust on the adaxial surface of the leaf. We infiltrated two regions of the maize leaf with AquaDust and subjected them to the following treatments using an optically transparent tape that is impermeable to gas exchange (low fluorescence optical transparent tape—Nunc Sealing, Thermo Scientific Inc., Waltham, MA): i) BT—Taping both sides of the leaf such that E_ab_ + E_ad_ ≈ 0, and ii) NT—No taping. On the first day, well-watered plants were probed. On two consecutive days, plants were sampled with a range of varying decreasing stem water potentials (increasing drought stress) above the turgor loss point in maize. Drought stress was imposed by withholding water on days two and three, leading to decreasing values of $\psi_{\mathrm{xyl}}$. For the data reported in Fig. 2, 87 measurements on 37 plants were performed for various values of $\psi_{\mathrm{xyl}}$ and VPD in an uncorrelated manner, as illustrated in *SI Appendix*, Fig. S2. After gas exchange measurements were taken, we measured the local adaxial leaf water potential ($\psi_{\mathrm{ssc}}^{\mathrm{apo}}$; $h_{\mathrm{ssc}}^{\mathrm{apo}}$) on the same leaf on which gas exchange was measured, with an optical point probe, and finally, the water potential at the BT location ($\psi_{\mathrm{xyl}}$; $h_{\mathrm{xyl}}$). With the data obtained in this manner, we calculate the effective OXZ conductance as

[1] $$g_{\mathrm{OXZ}}=\frac{E^{\mathrm{ad}}+E^{\mathrm{ab}}}{w_{\mathrm{xyl}}-w_{\mathrm{ssc}}^{\mathrm{ad}}}$$

The stomatal conductance was estimated from the equations in the LICOR LI6800 manual with the following modification: in the expression that represents the total conductance of a leaf to water vapor flux

[2] $$g_{s}=\frac{E(1-\frac{w_{i}+w_{a}}{2})}{w_{i}-w_{a}}$$

the value of $w_{i}$ (= $w_{\mathrm{ssc}}^{\mathrm{apo}}$) measured with AquaDust was used instead of the saturation vapor pressure at the leaf temperature, $w_{\mathrm{sat}}\left(T_{\mathrm{leaf}}\right)$. Further details of measurement procedures are explained in *SI Appendix*, section S3.

### Confocal Imaging.

Intact leaves from slac1-2 null mutants (W22 background) were infiltrated with AquaDust and imaged using a Zeiss LSM880 confocal microscope with a long working distance 20x objective (LMPlanFL N 20x/0.4, Olympus) while maintaining the leaves in a gas exchange cuvette (CIRAS-3, PPSystems). Two-channel imaging was performed using 488 nm and 561 nm excitation to collect AquaDust FRET signals and tissue autofluorescence. Water potential was calculated pixel-by-pixel from FRET efficiency using calibrated response curves. Cell morphology analysis was performed using ImageJ on autofluorescence images. Detailed imaging protocols, calibration procedures, and analysis methods are provided in *SI Appendix*, section S8.

## Supplementary Material

Appendix 01 (PDF)

## Data Availability

The MatLab files are all in the GitHub repository (39). All other data are included in the article and/or *SI Appendix*.
